# The Unbearable Indefiniteness of Spacetime

**DOI:** 10.1007/s10701-025-00819-4

**Published:** 2025-02-08

**Authors:** Enrico Cinti, Cristian Mariani, Marco Sanchioni

**Affiliations:** 1 Department of Philosophy, University of Geneva, 5, Rue de Candolle, CH-1211 Gèneve 4, Geneva, Switzerland; 2 Institute for Language, Logic, and Computation, University of Amsterdam, 1090 GL Amsterdam, The Netherlands; 3 Istituto di Studi Filosofici, Università della Svizzera Italiana, Via Giuseppe Buffi 13, 6900 Lugano, Switzerland; 4 Istituto Universitario Sophia, Via San Vito n.28, Loppiano, 50064 Figline e Incisa Valdarno, Florence Italy

**Keywords:** Loop quantum gravity, Non-commutativity, Ontological indeterminacy, Quantum gravity, Quantum indeterminacy, String theory

## Abstract

We consider the observables describing spatiotemporal properties in the context of two of the most popular approaches to quantum gravity (QG), namely String Theory and Loop QG. In both approaches these observables are described by non-commuting operators. In analogy with recent arguments put forward in the context of non-relativistic quantum mechanics [see Calosi and Mariani (Philos. Compass 16(4):e12731, 2021) for a review], we suggest that the physical quantities corresponding to those observables may be interpreted as *ontologically indeterminate*—i.e., indeterminate in a way that is non-epistemic and semantic-independent. This working hypothesis has not received enough attention in the current debate on QG, and yet it may prove explanatory useful in several respects. First, it provides a clear background for understanding how some features of QG are ontologically continuous to features of quantum mechanics. Second, it sets the stage for asking new interesting questions about QG, for instance concerning the status of the so-called Eigenstate-Eigenvalue link. Third, it indirectly shows how the debate on *ontological indeterminacy* may extend well beyond the non-relativistic case, contrary to what seems to be assumed. Fourth, and perhaps more importantly, it provides a promising alternative to the received view on QG [Wüthrich et al. (Philosophy Beyond Spacetime: Implications from Quantum Gravity, Oxford University Press, Oxford, 2021)] according to which spacetime is not fundamental. On the view we shall suggest, spacetime may be indeterminate and yet fundamental.

## Introduction

In any quantum theory there are specific pairs of physical quantities—the *non-commuting* ones—which cannot simultaneously be assigned a definite value. This feature is at the core of many discussions regarding the ontology of the theory and the conceptual implications of the formalism. So much so, that arguably such *lack of value definiteness* (LVD) is one of the marks allowing to distinguish quantum from classical theories.

A novel approach has emerged in recent years which attempts to interpret LVD in a systematic way and by recognising how pervasive this feature is. The idea is to take LVD at face value as indicating that the physical world is in some respect *objectively indeterminate*—i.e., it is indeterminate independently from our knowledge or from our representations of it. The existence of *ontological* indeterminacy—sometimes also called *metaphysical*—has been suggested within the context of the orthodox interpretation of quantum mechanics [1–5], the spontaneous collapse models [6], the Many-Worlds interpretation [7], Relational Quantum Mechanics [8], and the Modal Hamiltonian interpretation [9]. Common to all these proposals is the idea that we can individuate certain physical quantities that are crucial for describing the ontology of the theory (such as *position*, *spin*, or *mass density*), and show that they have to be interpreted as objectively indeterminate given the formal structure of the theory.

Although the discussion so far only focused on examples from non-relativistic quantum mechanics (see [10] for a review), the mathematical and conceptual features on which the existing arguments rely should extend to virtually every theory that has the right to be called *quantum*. In particular, the *non-commutativity* of the operator algebra appears to play a pivotal role in the whole debate [4, 11]. Based on this general consideration, in this paper we shall investigate the status of ontological indeterminacy and of LVD within two of the major approaches to quantum gravity (QG), namely loop quantum gravity (LQG) and string theory (ST) [12].

When it comes to LQG, the non-commutativity which interests us comes from attempting to reconcile the existence of a minimal area in the theory with the need for Lorentz invariance, which demands that all lengths vary continuously under Lorentz boosts. This reconciliation relies on Lorentz boosted operators not commuting with their unboosted counterparts. Regarding ST, non-commutativity is a consequence of the quantisation of the scalar fields living on the string’s worldsheet. We can then start by canonically quantising the scalar fields of the string, which can be treated as a type of position variable. Together with their associated conjugate momenta, one finds that these scalar fields form a non-commutative algebra of operators, satisfying non-commutativity relations essentially analogous to those of the well-known position/momentum case in quantum mechanics. The crucial point for this paper is that in both cases, the consistency of the theory relies crucially on the claim that certain operators cannot represent determinate quantities at the same time. These properties are in LQG the geometric properties encoded by the area operator, while in ST they are the centre of mass position, centre of mass momentum, and higher harmonic modes of the string. In LQG, we have an analogy with angular momentum, which makes particularly explicit the connection with analyses of ontological indeterminacy already developed in the existing literature. In ST, too, the fact that we are dealing with the non-commutativity of position and momentum operators is strictly analogous with the standard non-relativistic case.^1^ The crucial, structural difference between the cases already studied in the literature and that of QG, appears to be that in QG the relevant operators are directly describing spacetime itself, rather than its occupiers. This naturally makes one wonder whether the resulting picture is one where spacetime is indeterminate.

Relatedly, an important difference will concern how we can attribute physical meaning to these observables and to the properties involved. In the case of quantum mechanics, the standard way of assigning definite values to a given observable is through the Eigenstate-Eigenvalue Link [13], which roughly establishes a one-to-one mapping between definite properties and eigenstates. In the context of QG, however, the status of the EEL is yet to be fully understood. Even more importantly, we notice that the existing arguments for *ontological indeterminacy* based on the EEL show that certain properties are indeterminately instantiated *against the background of a classical spacetime*. In QG, instead, the fact that the resulting indeterminacy affects properties or structures of spacetime itself makes it much harder to understand how to correctly individuate indeterminate states or properties.

The received view in the discussion of the philosophical consequences of QG is that we are forced to abandon the idea that spacetime is fundamental, and embrace the thesis of an *emergent spacetime* [14]. An alternative to this view, which we will suggest towards the end of this paper, may instead be based on the thought that spacetime as described by QG is indeterminate (in a way that has to be specified) while still being considered fundamental.^2^

*Roadmap*. In Sect. 2 we introduce the standard argument for the existence of *ontological indeterminacy* in non-relativistic quantum mechanics (QM). We then present the cases for the existence of indeterminacy in Loop Quantum Gravity and String Theory respectively in Sects. 3.1 and 3.2. In Sect. 4 we draw some philosophical morals, in particular about the status of the Eigenstate-Eigenvalue Link Sect. 4.1, the nature of location Sect. 4.2, and the possibility of a fundamentally indeterminate spacetime in QG Sect. 4.3.

## Indeterminacy in Quantum Mechanics

The *lack of value-definiteness* (LVD) indicates that quantum observables do not possess definite values at all times. In order to have a better grasp on LVD, following the existing literature [10], we shall start by stating the general principle through which we can assign values to physical quantities based on the quantum state. In the standard formalism, this is done by the so-called Eigenstate-Eigenvalue Link (EEL),^3^ which can be stated as follows:

**EEL:** A physical system *s* has a definite value *v* of a given observable $\mathcal {O}$ *if and only if* *s* is in an eigenstate of $\mathcal {O}$.

Through the EEL, we can then give a classification of various cases where LVD arguably emerges. The most accurate such classification has been given by [4], who individuate three distinct features of the theory giving rise to LVD. These are (i) *Incompatible Observables*, (ii) *Superposition*, and (iii) *Entanglement*. As regards to (i), consider two observables $\mathcal {O}_1$ and $\mathcal {O}_2$. These observables commute *if and only if* $[\mathcal {O}_1,\mathcal {O}_2]=\mathcal {O}_1\mathcal {O}_2-\mathcal {O}_2\mathcal {O}_1$ = 0 . If they do not satisfy this constraint, they do not commute, and are called *incompatible*. Since incompatible observables do not share the same eigenstates, if the system *s* is in one such eigenstate of, say, $\mathcal {O}_1$, it follows that it does not have a definite value for $\mathcal {O}_2$ (and *viceversa*). As regards to (ii), note that a linear combination $|\psi \rangle =q_1|\phi _1\rangle +q_2|\phi _2\rangle$ of different eigenstates $|\phi _1\rangle$ and $|\phi _2\rangle$ of an observable $\mathcal {O}$ is not always an eigenstate of $\mathcal {O}$. If a system *s* is in $|\psi \rangle$, it follows that it does not have a definite value of $\mathcal {O}$. Finally, take (iii), *entanglement*. Consider a system $s_{12}$ composed by $s_1$ and $s_2$ with corresponding Hilbert space $\mathcal {H}_{12}=\mathcal {H}_1\otimes \mathcal {H}_2$. $s_{12}$ may be in an eigenstate $|\psi \rangle$ of $\mathcal {O}_{12}$ that is neither an eigenstate of $\mathcal {O}_1$ nor an eigenstate of $\mathcal {O}_2$—with $\mathcal {O}_1$ and $\mathcal {O}_2$ defined on $\mathcal {H}_1$ and on $\mathcal {H}_2$ respectively. From this it follows that $s_1$ and $s_2$ will lack a definite value for both corresponding observables. Although there are crucial conceptual differences between these three cases (see [4] for an extensive discussion), for what matters to us here the result of applying EEL to each of them is the same, namely that one or more observables do not always possess a definite value.

Defenders of *quantum indeterminacy*, such as [4], argue that LVD should be taken at face value as indicating that the world is *ontologically indeterminate*.^4^ Two distinct families of approaches have been proposed to account for this, which [22] calls *meta-level* and *object-level* views. According to the former, very roughly, indeterminacy is understood as worldly unsettledness between fully precise alternatives. On this view, ontological indeterminacy occurs whenever it is indeterminate which determinate state of affairs obtains. This view is meant to capture the phenomenon of indeterminacy *modally*, and in a way not dissimilar from how we account for other notions such as possibility or necessity. According to the *object-level* view instead, indeterminacy is understood as the (determinate) obtainment of an indeterminate state of affairs. The crucial explanatory component of this approach is then played by the definition of an indeterminate state of affairs, which can be given in various ways. The most discussed view, developed by [23], exploits the distinction between determinable and determinate properties. On this view, an indeterminate state of affairs is one where a given entity instantiates a determinable (e.g. *red*) without instantiating a unique determinate (e.g. *scarlet* or *crimson*). The non-uniqueness requirement can, in turn, be satisfied in at least three ways [24]: *gappy* has it that no determinate is instantiated; *glutty relativised* has it that more than one is instantiated, although each relative to something; *glutty degree* has it that more than one is instantiated, each with a certain degree *n*, with (*n* $< 1$).

The argument leading from LVD to the existence of quantum indeterminacy is not necessarily tied to the EEL. For instance, ontological indeterminacy may also arise in other interpretations of QM, which reject this link,^5^ However, it is common practice in the literature to start with the EEL in order to give the clearest explanation of the emergence of ontological indeterminacy [13]. We shall come back later on, in Sect. 4, to the issue of how, and to what extent, considerations about indeterminacy are tied to the EEL in the context of QG.

For now, it is important to focus on one important issue which has not received enough attention so far, and which however will prove crucial particularly for our discussion. In all the cases of indeterminacy in standard, non-relativistic QM, we are allowed to think that some state or property is indeterminately instantiated against the background of a classical spacetime. In other words, we could (at least in principle) distinguish a certain indeterminate state from another by simply *locating* it in spacetime. While it could be indeterminate *where* the object is located (in virtue of having an indeterminate position), it is always correctly assumed that the locations themselves, i.e. spacetime regions, behave classically and in a determinate way. It seems in fact plausible that the only way to grasp what it means for a microscopic object to have an indeterminate position, is by assuming that the locations (the positions that the object could occupy) are not themselves indeterminate,^6^ This situation will change radically in the context of QG, as we are about to see.

## Non-Commutativity in Quantum Gravity

Our primary goal in this section is to explore the appearance of ontological indeterminacy in QG by dint of a simple model naturally arising in Loop Quantum Gravity [27, 28] in Sect. 3.1, and by looking at the canonical quantization of the string in perturbative String Theory [29] in Sect. 3.2.

In particular, we will see that the indeterminacy emerging from these constructions is of the incompatible observables type, i.e. that two (or more) observables cannot have well-defined values simultaneously. This fact stems from a given system instantiating properties described by those operators that cannot be in an eigenstate at the same time.

### Loop Quantum Gravity

Let us first remark on one of the features that one might expect a quantum gravity theory to realise: discreteness of spacetime. In particular, we might expect that the spacetime described by a consistent quantum gravitational model will display a minimal length of some kind and that this minimal length coincides with the Planck length. By minimal length here, we mean a length such that no observer can measure lengths shorter than that.^7^ Thus, if we take the Planck length to be our minimal length, no observer is allowed to measure lengths shorter than the Planck length. However, such a minimal length might, prima facie, appear to contrast with Lorentz invariance,^8^ as we will see now. This apparent conflict between minimal lengths and Lorentz invariance and its resolution within models of LQG will be the origin of ontological indeterminacy in these LQG models.^9^

Let us start by briefly looking at why minimal length and Lorentz invariance might appear to be in contrast. It is helpful to start by remembering that one of the symmetry transformations of the Lorentz group are boosts,^10^ which take an observer in a reference frame at rest to an observer in a reference frame moving with constant velocity with respect to the first. Such boosts will usually take the following form:

1 $$\begin{aligned} t'= & \gamma (t - \frac{vx}{c^2}) \end{aligned}$$

2 $$\begin{aligned} x'= & \gamma (x - vt) \end{aligned}$$

3 $$\begin{aligned} y'= & y \end{aligned}$$

4 $$\begin{aligned} z'= & z \end{aligned}$$

where $\gamma = \frac{1}{\sqrt{1 - \frac{v^2}{c^2}}}$, (*t*, *x*, *y*, *z*) and $(t',x',y',z')$ are sets of coordinates in two different reference frames moving with relative velocity *v* in direction *x*, and *c* is the speed of light.

The critical feature of these transformations for this article is that they give rise to two of the most celebrated predictions of special relativity (which extend to any Lorentz invariant theory): length contraction and time dilation.^11^ In particular, what matters here is length contraction. By length contraction, we mean that, if we take a rod of length *l* in a given reference frame, upon moving to the reference frame of an observer moving at constant velocity *v* with respect to us, we find that our rod in this new reference frame is no more of length *l*, but of length $l' = \frac{1}{\gamma } l$. Thus, our rod got shorter when moving to a boosted reference frame.

With that being said, one can immediately see why Lorentz boosts, and thus Lorentz invariance, are not compatible with a minimal length. Take an observer in a given reference frame who measures a given length. Let us also stipulate that the result of their measurement is the minimal length *l*, which we identify with the Planck length $l_{Planck}$ for simplicity.^12^ Let us now move to a boosted reference frame. In this new reference frame, by length contraction, a boosted observer performing our original observer’s measurement will not get $l_{Planck}$ as a result, but $l' = \frac{1}{\gamma } l_{Planck}$, i.e. less than $l_{Planck}$. Thus, we have seen a way to measure a length shorter than the minimal length, which is, of course, incompatible with the definition itself of minimal length. Thus, Lorentz invariance and minimal lengths are incompatible.

The argument, as stated, is certainly less than conclusive and can be resisted at various points. Let us briefly mention only one: gravity is not (globally) Lorentz invariant, but only locally Lorentz invariant. While this fact is undoubtedly true, let us observe two points in response. (i) Violations of local Lorentz invariance would be troubling nonetheless, and in any case, (ii) one can reformulate the above scenario by taking a quantum gravitational spacetime whose classical limit is flat spacetime and considering small scale quantum effects in this classical background, which would then display the features mentioned above.^13^ Moreover, besides the various issues with the argument presented above, what is instructive is to see how, concretely, we can avoid situations such as those described above in the context of a theory of quantum gravity such as Loop Quantum Gravity. Note that nothing in this article depends on the air-tightness of the argument presented above, but only on the features of Loop Quantum Gravity that avoid its conclusion, which are independent of the argument’s assumptions.

Let us briefly recast the above scenario in a way more natural for Loop Quantum Gravity [37, 38]. Loop Quantum Gravity is an approach to quantum gravity that starts by recasting General Relativity in terms of a new set of variables (Ashtekar’s variables) which put General Relativity in a form close to *SU*(2) Yang-Mills theory. From this point of view, one then attempts to quantise gravity in the standard way that one would quantise any field theory. The result of this quantisation procedure is then Loop Quantum Gravity. While we will not be concerned with the details of Loop Quantum Gravity’s formulation, it is essential to note that in Loop Quantum Gravity, discreteness of spacetime quantities is most naturally seen in the discreteness of the spectra of the area and volume operators, rather than in an explicit minimal length. We will thus focus on area operators, though note that nothing substantial changes from the discussion in terms of a minimal length. The spectrum of the area operator $\mathcal {A}$ is the following:

5 $$\langle A\rangle = 8\pi \gamma l_{Planck}^2\sqrt {j(j + 1)} ,$$

where $\langle A \rangle$ is an area eigenvalue, $\gamma$ is a free parameter,^14^$l_{Planck}$ is Planck’s length and *j* are irreducible representations of the group *SU*(2), which means that $j=0,\frac{1}{2},1,\frac{3}{2},2,\dots$. It is immediate to see that the spectrum of the area operator $\mathcal {A}$ is discrete since the *j*s, being representations of *SU*(2), are discrete. This fact also implies that there is a minimal area, corresponding to the smallest non-zero eigenvalue of $\mathcal {A}$.

To get our minimal length problem, one can repeat the steps described above and consider what a Lorentz boosted observer would observe if they were to measure the area. Naively, we would expect a contraction of the area and a conflict between Lorentz invariance and minimal area in Loop Quantum Gravity. This description, however, is not the whole story. Let us be slightly more precise about what is going on in this scenario. An observer is measuring the area operator $\mathcal {A}$, thus projecting into an eigenstate of $\mathcal {A}$.^15^ They then compare their result with that of a second observer, who measures a different operator, $\mathcal {A'}$, which is the result of applying a Lorentz boost to $\mathcal {A}$. Now, this comparison is straightforward only if $\mathcal {A}$ and $\mathcal {A'}$ commute, otherwise we cannot compare the two since they would not have well-defined values at the same time because they would not have eigenstates in common. Indeed, as shown in [27], $\mathcal {A}$ and $\mathcal {A'}$ do not commute:

6 $$[\mathcal {A},\mathcal {A'}] \ne 0~.$$

Thus, when one measures $\mathcal {A}$ and then tries to see if $\mathcal {A'}$ will show a contracted area smaller than the minimal one by length contraction, their efforts will ultimately crash against the fact that $\mathcal {A'}$ does not show a well-defined value for the area since the system is not in an eigenstate of $\mathcal {A'}$. Thus, it does not make sense to ask what is the area measured by the observer measuring $\mathcal {A'}$, as there is no definite answer to this question since we find a superposition of eigenstates of $\mathcal {A'}$^16^ whenever we observe an eigenstate of $\mathcal {A}$. While $\mathcal {A}$ and $\mathcal {A'}$ have the same spectrum, eigenstates of $\mathcal {A}$ correspond to superpositions of eigenstates of $\mathcal {A'}$. In this way, within Loop Quantum Gravity, we avoid the apparent conflict between a minimal area and Lorentz invariance. Speaking somewhat operationally, in the boosted frame, one measures a statistical distribution of discrete eigenvalues of the area rather than a single contracted eigenvalue.

Interestingly enough, as observed by [27], this result relies essentially^17^ on the same mechanism by which quantised angular momentum is compatible with invariance under rotations in Quantum Mechanics. In both cases, it is the non-commutativity of certain operators connected by the relevant symmetry that allows us to implement the symmetry and preserve their spectrum at the same time by making it impossible to have an eigenstate for the two operators at the same time. Without a single eigenstate for both operators, the symmetry-related operators (for example, by Lorentz boosts) will not both have meaningful information about areas at the same time, let alone about an area smaller than the minimal one. Since, however, the claim that Lorentz boosts and minimal area were in tension relied crucially on the comparison between what an operator at rest and one Lorentz boosted could tell about area, the statement of this tension is itself impossible.

It is immediate now to see the connection with ontological indeterminacy. Since incompatible observables are formally represented as non-commuting operators, and since incompatible observables are taken to be one of the main sources of ontological indeterminacy in QM, it seems that also in this case coming from LQG we may speak of the existence of some ontological indeterminacy. However, before moving to consider the application of ontological indeterminacy to quantum gravity more in detail, let us consider a second example of the appearance of ontological indeterminacy in quantum gravity, this time coming from string theory. We will do this in the next section.

### String Theory

Let us now turn our attention to String Theory. Before starting, let us make two technical disclaimers for the ensuing discussion. As with any quantum field theory, string theory has two regimes: perturbative and non-perturbative.^18^ After the development, on one side, of holography and AdS/CFT [39], and, on the other, of M-theory [40] and F-theory [41], much progress has been made in the non-perturbative regime of String Theory, which, however, remains far from being satisfactorily under control. In this section, then, we will focus on the perturbative sector of String Theory, the so-called *perturbative String Theory*, which is, by now, well-known.^19^ Moreover, for ease of exposition, we will concentrate just on Bosonic String Theory. The extension of our discussion to Superstring Theory is straightforward.^20^ Let us also remind the reader that the concrete example of indeterminacy that we study in this section is chosen for simplicity but is far from being the only possible one in String Theory.^21^

The fundamental objects of perturbative string theory are one-dimensional (closed and open) strings. As the dynamics of a point particle can be represented via its one-dimensional *world-line*, the dynamics of a string can be represented via its two-dimensional *worldsheet*, which we call $\Sigma$. The action $\mathcal S$ of perturbative string theory, called the Polyakov action, is a generalisation of the action of a point particle and can be written as follows^22^:

7 $$\begin{aligned} \mathcal S_{Poly} \left[ h,X\right] = \frac{1}{L_s^2} \int d^2 \sigma \sqrt{-h}~h^{\alpha \beta } \partial _\alpha X^\mu \partial _\beta X^\nu g_{\mu \nu } \left( X^\rho \right) ~, \end{aligned}$$

where $L_s$ is the string length, $\sigma ^0 = \tau$ and $\sigma ^1= \sigma$ are the world-sheet coordinates, $h^{ \alpha \beta }$ and *h* are respectively the inverse metric and the determinant of the world-sheet metric $h_{ab}$, which describes the geometry of the world-sheet. $X^ \mu \left( \sigma \right)$ is a map between the string world-sheet and the target space, i.e. the spacetime in which the string propagates, while $g_{ \mu \nu } \left( X \right)$ is at the same time the coupling constant of the string interactions and the metric of target space Here’s the revised phrase with the detailed discussion incorporated:

8 $$\begin{aligned} \mathcal S_{Poly} \left[ h,X\right] = \frac{1}{L_s^2} \int d^2 \sigma \sqrt{-h}~h^{\alpha \beta } \partial _\alpha X^\mu \partial _\beta X^\nu g_{\mu \nu } \left( X^\rho \right) ~, \end{aligned}$$

where $L_s$ is the string length, $\sigma ^0 = \tau$ and $\sigma ^1= \sigma$ are the worldsheet coordinates, $h^{\alpha \beta }$ and $h$ are respectively the inverse metric and the determinant of the worldsheet metric $h_{ab}$, which describes the geometry of the worldsheet. $X^\mu \left( \sigma \right)$ is a map between the string worldsheet and the target space, i.e., the spacetime in which the string propagates, while $g_{\mu \nu } \left( X \right)$ serves both as the coupling constant of string interactions and as the metric of the target space. In this context, $g_{\mu \nu }$ represents the target metric, typically expressed as Minkowski plus perturbations in perturbative string theory. From the worldsheet perspective, this metric also acts as a coupling constant in the sigma model framework, determining the interactions within the theory. This dual interpretation stems from the sigma model structure, where fields defined on the worldsheet map into the target space, and the dynamics encode information about the geometry of the target space. In perturbative formulations, the metric $g_{\mu \nu }$ is treated as a field in the target space and (often) expanded around a simple reference configuration, such as flat Minkowski spacetime, with small perturbations added. As a result, $g_{\mu \nu }$ can be thought both as the spacetime geometry and as a coupling constant for interactions in this setup.

The Polyakov action (8) has, *prima facie*, two degrees of freedom, namely the worldsheet metric $h_{\alpha \beta }$ and the embedding coordinates $X^\mu$. Moreover, the action (8) has two local symmetries (diffeomorphisms and Weyl symmetry) and one global symmetry, inherited by target space (Poincaré invariance). In order to make sense of the quantum mechanical description of (8), i.e. in order to define a path integral, write down correlation functions and compute scattering amplitudes, we need to fix a gauge for the local symmetries. A complete review of the construction of the path integral for the Polyakov action (8) goes beyond the scope of this paper. We limit ourselves to the result of the quantisation procedure. In order to write down a path integral for theories with local symmetries, one first needs to gauge fix. Since the worldsheet metric can be completely gauge fixed, it does not have any propagating degrees of freedom, and thus it is not part of the physical content of the theory.^23^ The upshot of the quantisation procedure is that one trades the path integral over the metric $h_{\alpha \beta }$ with a path integral over the new (ghost) fields $b,~\bar{b}, ~c$. One can write correlation functions of *N* (gauge-invariant) operators in the following way:

9 $$\begin{aligned} \langle \langle V_1\dots V_N\rangle \rangle= & \int \frac{\mathcal D X^\mu ~\mathcal D h_{\alpha \beta }}{\text{ Vol }\left( \text{ gauge }\right) }\left( V_1\dots V_N \right) e^{-S_{Poly} \left[ h,X\right] } \end{aligned}$$

10 $$\begin{aligned}= & \int \mathcal D X^\mu ~\mathcal D b ~\mathcal D \bar{b} ~\mathcal D c\left( V_1\dots V_N \right) e^{-S_{matter} \left[ X\right] - S_{ghost}\left[ b,\bar{b}, c\right] } \end{aligned}$$

where $S_{matter} \left[ X\right]$ is the *matter action* and $S_{ghost}\left[ b,\bar{b}, c\right]$ is the *ghost action*.

Let us now review the quantisation of the space of solutions of the gauge-fixed Polyakov action, focusing on the matter action $S_{matter} \left[ X\right]$.^24^ By a proper choice of gauge, the so-called conformal gauge,^25^ one can write the equation of motion derived from $\mathcal S_{\text{ matter }}$ in the following way:

11 $$\begin{aligned} \partial \bar{\partial }X^\mu = 0 \end{aligned}$$

where $\partial$ and $\bar{\partial }$ are derivatives with respect to $\omega$ and $\bar{\omega }$ respectively. A general solution of (11) can be written in the form $X^\mu (\omega ,\bar{\omega }) = X_L^\mu (\omega ) +X_R^\mu (\bar{\omega })$, where $X_L^\mu (\omega )$ and $X_R^\mu (\bar{\omega })$ are respectively left and right moving modes of the string, and are holomorphic functions of $\omega$ and $\bar{\omega }$. There are two types of fundamental objects in perturbative String Theory, open and closed strings, each requiring a solution of (11); for simplicity we only look at closed strings.

**Fig. 1:**
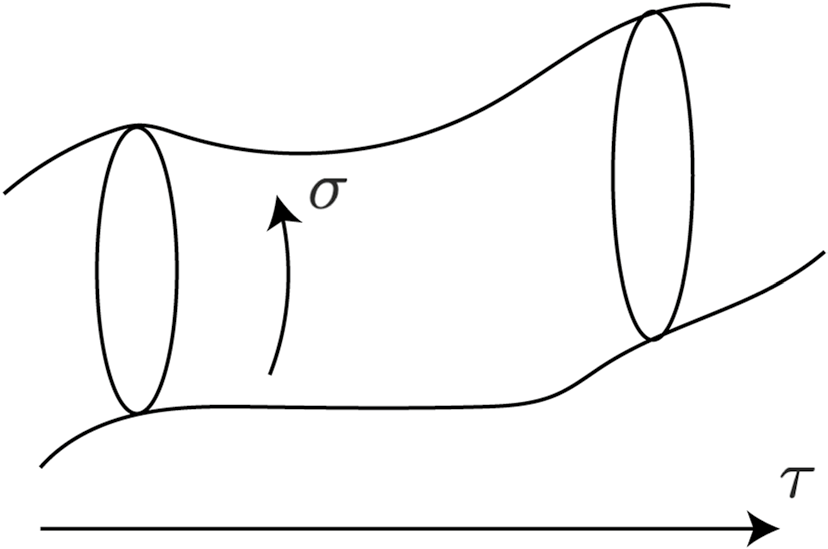
A schematic picture of a closed string propagating in spacetime. $\sigma$ represents the periodic spatial coordinate of the worldsheet, while $\tau$ represents its time coordinate

**Closed strings** (Fig. 1) are defined by asking periodicity on $\omega$ and $\bar{\omega }$, i.e. $X^\mu \left( \omega + 2\pi i, \bar{\omega }-2\pi i\right) = X^\mu \left( \omega , \bar{\omega }\right)$.

A general solution for left and right moving modes of the string can be written in the following form

12 $$\begin{aligned} X_L ^\mu (\omega )= & \frac{1}{2}X_0^\mu -\frac{i}{2}\alpha ' P^\mu \omega + i \sqrt{\frac{\alpha '}{2}}\sum _{n\ne 0} \frac{\alpha _n}{n}e^{n\omega }\end{aligned}$$

13 $$\begin{aligned} X_R^\mu (\omega )= & \frac{1}{2}X_0^\mu -\frac{i}{2}\alpha ' P^\mu \bar{\omega }+ i \sqrt{\frac{\alpha '}{2}}\sum _{n\ne 0} \frac{\bar{\alpha }_n}{n}e^{n\bar{\omega }} \end{aligned}$$

which are chosen in order to solve the equation of motion (11) and obey the periodicity condition. As common in analytic mechanics, one can also write down the momentum density, i.e. the conjugate variable of $X^\mu$^26^

14 $$\begin{aligned} \mathcal {P}_\mu = \frac{i}{2\pi \alpha '} \left( \partial X_\mu +\bar{\partial }X_\mu \right) \end{aligned}$$

Having $X^\mu$ and $\mathcal P_\mu$, one can apply the rules of canonical quantisation, i.e. demand that $\left[ X^\mu (t,\sigma ), \mathcal P_\nu (\tau ,\sigma ')\right] = i~ \eta ^\mu _\nu ~\delta \left( \sigma -\sigma '\right)$. Using the explicit expansions of $X^\mu$ and $\mathcal P_\mu$ given by Eqs. (12,13) and (14), one gets the following commutation relations for the expansion coefficients of the $X^\mu$s:

15 $$\begin{aligned} \left[ X_0^\mu , P^\nu \right]= & i~ \eta ^{\mu \nu } \end{aligned}$$

16 $$\begin{aligned} \left[ \alpha _n^\mu , \alpha _m ^\nu \right]= & n~ \eta ^{\mu \nu } \delta _{n+m} \end{aligned}$$

17 $$\begin{aligned} \left[ \bar{\alpha }_n^\mu , \bar{\alpha }_m ^\nu \right]= & n~ \eta ^{\mu \nu } \delta _{n+m}\end{aligned}$$

18 $$\begin{aligned} \left[ \alpha _n^\mu , \bar{\alpha }_m ^\nu \right]= & 0 \end{aligned}$$

The Hilbert space of the theory for closed strings is $\text{ Span } \left\{ \alpha _{-n_1}^{\mu _1} \dots \alpha _{-n_k}^{\mu _k}\bar{\alpha }_{-m_1}^{\nu _1} \dots \bar{\alpha }_{-m_q}^{\nu _q}\right\} |0,p\rangle$.^27^

We are interested in a physical interpretation of the commutation relations (15)–(18). For instance, in the closed string expansion (12,13)^28^ ...$X_0^\mu$ represents the centre of mass position of the string, $P_\mu$ represents its centre of mass momentum, while $\alpha$ and $\bar{\alpha }$ represents its higher harmonic modes. The commutation relations (15)–(18), thus, entail that there are non-trivial commutation relations between these observables.

Again, as in Sect. 3.1, we see that the appearance of the non-commutativity of certain observables leads to non-trivial commutation relations, which can be naturally understood, as we have learned in QM, as meaning that certain observables are incompatible. Some comments regarding the spacetime interpretation of these commutation relations, however, are in order. Indeed, one might worry that we have discussed thus far shows at most that the position of the string in spacetime in indeterminate, not the string itself; this observation would render the string theory case much different from the LQG from Sect. 3.1. This point can be strengthened by the fact that we are dealing with perturbative string theory, and so a field like $X_0^\mu$ will give perturbative corrections to a classical metric, in a perturbative expansion around some GR background for string theory. These perturbative corrections are naturally identified with the dynamics of string propagating in such a background.

In response to this objection, note, first of all, that the commutation relations (15)–(18) are exact relations obtained by canonical quantization; as such, no perturbation theory entered into obtaining them, and hold exactly on the Hilbert space of string theory. In particular, we do not need a split between a (perturbative) background and some perturbations to obtain (15)–(18). Hence, the $X_0^\mu$ field appearing in them should not be understood as simply encoding the dynamics of a string propagating in spacetime, but rather the whole (quantized) spacetime metric. Perturbation theory enters into this picture because we can evaluate the expressions presented in this section only perturbatively; however, this does not mean that the expression themselves depend substantially on perturbation theory in a problematic way for our arguments. Indeed, given that we have obtained them through canonical quantization, they do not.

Relatedly, even if perturbation theory was necessary to make sense of expressions like (15)–(18), our discussion of spacetime indeterminacy in string theory would remain unaffected. Indeed, even in perturbation theory, in a theory of QG like string theory one is still computing spacetime properties. For example, when computing quantum corrections to correlation functions of gravitons, which are straightforwardly computed in perturbative string theory, one is computing quantum properties of spacetime via strings, not of strings propagating in spacetime; just like in quantum field theory, by computing quantum corrections to scattering amplitudes, one is computing quantum properties of the quantum field, not of a particle propagating in that field’s background.^29^ Since such quantum corrections are obtained by a perturbative expansion of (9,10) in terms of the $X_0^\mu$ fields, then these fields encode quantum properties of spacetime also in perturbation theory. Hence, their commutation relations (15)–(18) encode indeterminacy for spacetime observables, just like in the case of LQG discussed in Sect. 3.1. To clarify, the operators $X^\mu$ and $P_\mu$ in the context of string theory play a dual role typical of sigma models. On the worldsheet, $X^\mu$ represents the fields mapping the worldsheet coordinates to the target spacetime, while $P_\mu$ corresponds to their conjugate momenta. These operators are fundamentally defined on the worldsheet but are interpreted as encoding physical properties of the string in the target space. Specifically, $X^\mu$ describes the position of the string, and $P_\mu$ describes its momentum in the target spacetime. In sigma models, this dual perspective highlights the intricate relationship between the worldsheet and target spacetime: the dynamics on the worldsheet encode geometric and physical information about the target space. This interplay is particularly significant in string theory, where the commutation relations between $X^\mu$ and $P_\mu$ reflect a quantum indeterminacy that is inherited by spacetime observables in the target space. This is analogous to the role of fundamental commutators in loop quantum gravity (LQG), where quantum operators for geometric observables encode a similar type of indeterminacy.

In the next section, we will look more in detail at some of the metaphysical implications of this connection.

## Philosophical Consequences of Spatiotemporal Indeterminacy

The two cases we presented rely on the appearance of non-commutativity to show that specific physical quantities cannot jointly be assigned a definite value in QG. These quantities, to recall, are represented by the Lorentz boosted and unboosted area operators to account for the relation between minimal area and Lorentz invariance (in LQG), and by the position, momentum, and higher harmonic modes operators (in ST). Given the structural analogies between these cases and the standard case of ontological indeterminacy in QM (discussed in Sect. 2), we are now in the position to evaluate the status of ontological indeterminacy in QG as well.

### Indeterminacy and the EEL in Quantum Gravity

Consider that the standard quantum mechanical argument for indeterminacy heavily relies on the eigenstate-eigenvalue link (EEL). So, for a similar line of reasoning to be applied here, we should first of all establish whether something like the EEL can be defended in QG. To the best of our knowledge, there is no explicit discussion of the EEL in QG.^30^ Nonetheless, two main considerations seem to suggest that the EEL is both (i) preserved and (ii) preserved in its standard form. As for (i), notice that the primary role of the EEL is to map the abstract formalism onto physical states so as to understand how these can be empirically detected.^31^ Therefore, it is natural to expect that empirical adequacy would require assuming something like the EEL.^32^ As for (ii), approaches to QG are usually meant to be developed in continuity with standard QM, at least when it comes to basic interpretative tools of the quantum formalism such as the EEL. To be clear, of course, many things change in the formalism even when we move to QFT, and so it is natural to expect things to get even worse in the context of QG. However, our claim here is that theories of QG are developed building on quantum theory* as we find it in the textbooks*. Hence there is no reason to believe (unless we have independent reasons to do so) that a modified link would be needed. For these reasons, we take it that the acceptance of the EEL is a safe assumption. Indeed, one might even argue, in the LQG case, that (something like) the EEL is necessary for the argument presented in Sect. 3.1, since the boosted and unboosted quantities cannot have determinate values at the same time. Indeed, [27, p. 2] are explicit in claiming that “the theory predicts that, for [a boosted observer], the surface does not have a sharp area”, which not only seems to indicate a commitment to the EEL, but even suggests that the relevant physical quantities are interpreted as lacking a definite value. With all this being said, we should be very clear that the arguments that follow must be taken as heavily conditional on the assumption that the EEL is part of QG, and that no full defense of this assumption will be given here.^33^

The conclusion to be drawn is that it seems quite natural to work on the assumption that *something like* the EEL is preserved in QG. Obviously, and this has to be recognised, the expression *something like* is doing a lot of heavy lifting here. For one, notice that, at least *prima facie*, the standard EEL is written in such a way as to speak of quantities which can be directly measured (*resolved by direct observation*, one would say). In the cases we considered, it is unclear whether the relevant quantities can be measured even in principle. This difference entails an even more crucial disanalogy: the standard version of the EEL can be given a sort of operationalist reading, whereas the QG version (whatever it ends up being) hardly can. While we believe that there is much more to be said on this topic, for now we just stress that it is with no doubt a positive consequence of our discussion to highlight the need to reflect on the status of the EEL in QG.

Let us now comment on the relevance of these constructions for ontological indeterminacy. The crucial point for this article is that both in LQG and ST, the consistency of the quantum theory of gravity relies crucially on the claim that certain operators cannot all represent determinate properties at the same time. This situation is the same as that of ontological indeterminacy from incompatible observables described in Sect. 2. We have two properties and a physical theory that forces one of them to be indeterminate whenever the other is determinate. As usual in any quantum theory, the incompatibility of these properties is encoded in the non-zero commutator between the operators representing them. Indeed, in LQG, we even have an explicit analogy with angular momentum, which makes particularly evident the connection with analyses of ontological indeterminacy developed in quantum mechanics. In ST, the fact that we are dealing with the non-commutativity of position and momentum operators is immediately analogous with the standard quantum mechanical case. In QM, this kind of non-commutativity is precisely the type of phenomenon falling under the rubric of incompatible observables.

All that we need is the extension of this claim to quantum gravity, and the structural similarity between the two cases suggests that this extension should be seriously considered. Indeed, in all these cases, one is relying only on the non-commutativity of certain observables, coupled with EEL. Moreover, we interpret this non-commutativity in the same way, namely as the impossibility of instantiating two properties determinately at the same time. Thus, it looks like a refusal to treat the quantum gravity case as instantiating incompatible observables indeterminacy appears at best misguided, at worst inconsistent with the attitude taken towards the quantum mechanical case, be it that of angular momentum or that of position and momentum. It would appear from these considerations that refusing to accept the analogy between QG and QM *vis á vis* quantum indeterminacy would require a principled reason as to why the two cases differ. However, such a principled reason cannot be found within the mathematics of the two theories, since in these respects, they are perfectly analogous. Whence the disanalogy then? One possibility might be the denial of EEL in QG but not in QM. Such a move, however, would be a restatement of the problem of finding a principled disanalogy between QG and QM in this context. Indeed, in both cases, we are ultimately dealing with non-commutative algebras of operators, and EEL is just a (natural) way of associating determinate properties to such a structure. While the specific nature of the algebras of operators involved changes from QG to QM, all that is relevant for the arguments in favor of the existence of ontological indeterminacy is that they are non-commutative algebras of operators. For example, in QG, this algebra would presumably involve a diffeomorphism invariant collection of operators not carrying any global symmetry^34^; in QM, on the other hand, the relevant algebra is given by the bounded operators on some Hilbert space $\mathcal {H}$ carrying some representation of the global symmetry group of Galilean spacetime, the Bargmann group [47]. However, in both cases, the two algebras are ultimately non-commutative algebras of operators. Thus, it appears that absent some further (most likely extra-mathematical) principled reason for rejecting the analogy between QM and QG when it comes to quantum indeterminacy, we have to admit that QG involves metaphysical indeterminacy, at least to the extent that QM does.

At the end of the day, however, these considerations stand and fall with the acceptance of the argument from non-commutativity of observables to ontological indeterminacy. However, our goal here was not to show that ontological indeterminacy is inevitable in quantum gravity but only that it is *as natural as it is in QM*. Indeed, the two cases share some crucial features that allow us to extend the quantum mechanical analysis of indeterminacy to quantum gravity. It is this goal we claim to have reached. We believe this is important in itself, at least to the extent that it indicates a promising way of showing how certain features of the ontology of QG can be interpreted in continuity with respect to features of QM (an hope recently expressed by [48]).

### Indeterminate Locations

Let us now look at an aspect where QM and QG, at least in the examples we have considered here, diverge in how they instantiate metaphysical indeterminacy. As we discussed in this section, and reviewed more extensively in Sect. 2, non-commutativity, along with the EEL, entails that certain physical quantities are indefinite. In the case of QM, the relevant quantities can be given, at least to some extent, a clear physical meaning. This is not as straightforward for QG, for as we discussed, the quantities are meant to represent the geometrical structure of spacetime. While it could be accepted that a particular object possesses an indeterminate location, it seems much harder to believe that the *regions* themselves are indeterminate. Nevertheless, despite its counterintuitive nature, both approaches to QG discussed in this paper support this claim (in quite different ways). This fact seems to suggest that the possibility of spacetime being fundamentally indeterminate has to be taken seriously. It is interesting, to better make sense of this connection, to compare the situation between QM and QG concerning location and indeterminacy. As we said above, there are arguments in QM that purport to show that location can sometimes be indeterminate [21, 49, 50]. In particular, Ref. [49] shows that if anything, what is indeterminate in QM is the *exact location* of a given quantum system. By exact location here, we mean the region of spacetime occupied by a specific object and only by it. It is to be contrasted with *weak location*, which is any region of spacetime that is occupied by a given object, even if not entirely (there might be multiple objects located there that do not overlap). Since quantum systems can have indeterminate positions (the system can fail to be in an eigenstate of the position operator), there can be indeterminacy in their exact location in the sense that it would be indeterminate which region they exactly occupy. Equivalently, indeterminacy in QM affects only the properties of matter and not those of spacetime. QM speaks about spacetime at most derivatively, in the sense that certain properties of matter (such as indeterminacy of position) lead to certain peculiar phenomena in the relation between matter and spacetime (such as indeterminacy of location). On the other hand, QG describes spacetime directly, and not merely as a background for the representation of the properties of matter. Thus, indeterminacy in this context turns out to be more challenging to conceptualise.

Therefore, the situation in QG is more radical than it is in QM: it is not simply the location of a quantum system in a given region that is indeterminate, but rather it is the region itself which involves quantum indeterminacy. Thus, it seems that spacetime too can be said to be indeterminate,^35^ which leads to standard theories of location not being well defined in the first place (a point suggested by the literature on spacetime emergence in QG [52]). To see this point clearly, consider that location is understood as a relation between systems and (determinate) spacetime regions [53]. In classical mechanics, this function is a 1–1 mapping between systems and their exact locations. In QM, exact locations are indeterminate. We can, however, recover a notion of location as a 1–1 mapping if we relax our definitions, and instead of exact location, we use weak location. In QG, however, even this generalisation fails, and for radical reasons: since spacetime itself is indeterminate, there cannot be a well-defined function from quantum systems to spacetime regions of any kind since its range (spacetime) is not well-defined and thus unsuitable for the task. To put things differently, if spacetime regions are not well-defined (because they are indeterminate), then standard theories of location, which assume the determinacy of regions spacetime,^36^ are not adequate anymore. Instead, to make sense of location in QG one would need an entirely new theory that is designed to deal with indeterminate spacetime regions.^37^

### Indeterminate vs. Emergent Spacetime

We have seen that indeterminacy in QG immediately leads to some of the most puzzling aspects of QG, such as spacetime emergence.^38^ In a way, this fact was to be expected. Quantum indeterminacy is considered by its advocates to be a crucial feature of QM and to be at the heart of the many puzzles of the theory; given the similarity between QM and QG in this respect, it is only natural that indeterminacy, if it were to show up in QG, would show up centre-stage. As we mentioned, indeterminacy comes from the non-commutativity of certain quantum observables. This non-commutativity is controlled by the parameter $\hbar$, Planck’s constant. This parameter, however, also controls the strength of quantum effects: $\hbar = 0$ means classical physics.^39^ Thus, when dealing with QG, where the quantum effects concern spacetime, regimes with non-zero $\hbar$ correspond to those where spacetime is expected to be quantum and to ultimately disappear. However, these are also the regimes where the non-commutativity of quantum operators is manifest, leading to ontological indeterminacy. Thus, it is quite natural to conclude that *spacetime emergence* and *spacetime indeterminacy* are naturally connected in QG.

We can make this point even clearer by thinking in terms of decoherence, i.e. the process by which, starting with a pure state in a superposition, unitary evolution leads to a state which is to a very good approximation a probability distribution, represented by a density matrix, over the configurations entering the superposition state.^40^ Decoherence, then, can be naturally thought as the physical process embodying the transition from the quantum to the classical regime; hence, decoherence gives us the concrete physical process underlying the formal discussion of classical limits and $\hbar = 0$ given above. In particular, the configurations over which decoherence gives a probability distribution will be in general *quasiclassical* states, i.e. states for which incompatible observables are both specified to the highest precision possible given the uncertainty relations. In particular, then, these are states where indeterminacy is minimized, or better yet, states where determinacy is approximately realized. Hence, decoherence also gives us a model of how determinacy can emerge from indeterminacy. Moreover, given that in both cases the process is the same, decoherence shows even more clearly how approaching the classical limit also means removing indeterminacy. Since, as we remarked, in QG the classical limit corresponds to the limit where spacetime appears, we can see how decoherence naturally underpins our claim that spacetime emergence and quantum indeterminacy are naturally connected, since they are mediated by the same physical process, i.e. decoherence.

Let us start from a straightforward observation: it appears that most arguments for the disappearance of spacetime in QG rely on an implicit premise that spacetime, whatever it is, must be free of indeterminacy. This premise seems operative in observations such as those in [14], where the appearance of non-commutativity, and hence fuzziness, in the description of spacetime, is heralded as one signal of the breakdown of spacetime itself. More generally, the common claim that the target of spacetime emergence is the recovery of a 4 dimensional smooth Lorentzian manifold, i.e. a fully determinate, classical entity, seems to suggest that spacetime, whatever it is, must be something free of indeterminacy.

The connection between determinacy and spacetime becomes even clearer by noting how, for example, spin networks, the building blocks of LQG, are hardly non-spatiotemporal classical entities, i.e. before quantization. Classically, a spin network is just the (dual of) a triangulation of spacetime; while a triangulation of a manifold is not the same as the manifold itself, it would seem quite hard to justify why the triangulation would count as less spatiotemporal than the manifold from which it comes. To really see the non-spatiotemporal nature of spin networks, we need to quantize them, which leads to the geometric variables associated with them non-commuting, losing a straightforwardly geometric, and hence spatiotemporal interpretation. However, as we have discussed at length, the appearance of non-commuting variables, or incompatible observables, is nothing more than the appearance of indeterminacy, i.e. the failure of spin networks to be determinate. Hence, determinacy and spacetime appear to go together, much like their contrast pair, indeterminacy and disappearance of spacetime.

Indeed, the interpretation that we have offered in this paper for the appearance of non-commutativity in the description of spacetime, and in particular in the appearance of incompatible observables, is that these features should be interpreted in terms of ontological indeterminacy. The contrast here is immediate: spacetime emergence, insofar as it is regarded as a conceptually revisionary program, claims that in QG we deal, at the fundamental level, with non-spatiotemporal entities from which spacetime should emerge. In the indeterminacy approach we are discussing, instead, spacetime never really disappears, but rather loses its fully determinate status, and becomes an indeterminate entity. Given the quantum nature of the indeterminacy under discussion, it seems appropriate then to speak of *quantum spacetime* for this indeterminate structure, in analogy to how we speak of quantum fields for the quantum indeterminate counterparts of determinate classical fields.

This approach presents a variety of attractive conceptual features: first of all, it establishes a fundamental continuity between the concepts used in QG and those used in General Relativity; in particular, no *hard problem* of spacetime emergence [61] is present in this context, since both at the fundamental and at the classical level we are employing spacetime concepts, only at the fundamental QG level they appear to be indeterminate. Relatedly, an important advantage of this approach to the ontology of spacetime in QG is that it reduces the issue of recovering spacetime from QG to the issue of studying the classical limit of a quantum theory; and while this is a formidable conceptual issue, it is one that seems to us to be under much better control than issues of spacetime emergence.^41^

Finally, the indeterminate spacetime hypothesis allows us to make sense of claims along the lines of [38, pp. 146–147]’s claim that, in LQG:

The quanta of space described by the spin network states [...] should not be thought of as quanta moving in space. They are not *in space*. They *are* themselves physical space.

For in this context, it is straightforward to see how spin networks, or the strings of perturbative string theory, make up spacetime: it is the same way in which quantum mechanical degrees of freedom make up quantum systems in QM. For example, in LQG the connection between spacetime and spin networks goes through the fact that spin networks are eigenstates of the area operator, hence making up an (ontologically indeterminate) property of quantum spacetime, which, upon taking the classical limit, corresponds to the classical, determinate property of having a certain determinate area. In this sense, this story is perfectly analogous to the angular momentum story that [27] use as analogy for the QG case; moreover, a broadly analogous story can be told for the degrees of freedom of perturbative string theory and how they relate to classical spacetime. Contrast this straightforward story with the more complicated story that the advocate of spacetime emergence has to tell: in that case, the spin networks or the perturbative strings make up a non-spatiotemporal entity, whose properties have any relation to spacetime properties only insofar as they stand in an appropriate relation to the emergent spacetime. In other words, in this context, the area operator of LQG, or the $X^\mu$ fields of perturbative string theory, do not really represent any area, and are rather just operators in the Hilbert space of LQG; we think of them in terms of areas or positions only because, when we move to a regime where we can identify an emergent spacetime, the properties described by the area operator or the $X^\mu$ fields make up, in some sense to be determined (and indeed object of intense debate, see, e.g., [62]), the classical areas or positions described by general relativity. This picture appears to us to be at least as conceptually cumbersome as the one offered by the indeterminate spacetime hypothesis, if not significantly more cumbersome.^42^ While we have only sketched the basics of such an account, we take it that the indeterminacy approach to the ontology of spacetime in QG provides a new and exciting avenue to study the conceptual foundations of QG.^43^

## Conclusions

The main goal of this paper has been to argue that there is a profound conceptual continuity between QM and QG when it comes to understanding the ontology of the so-called *lack of value definiteness* for physical quantities. We have shown that in two of the most developed approaches to QG, namely Loop Quantum Gravity and String Theory, the observables representing the geometric structure of spacetime are non-commuting ones. We have argued that these quantities can be considered indeterminate by building upon the standard reasoning leading to ontological indeterminacy in standard QM. These results point towards the possibility that, according to theories of quantum gravity, spacetime is indeterminate and yet fundamental. This conclusion has various consequences that are worth developing further. First, it provides a clear background for understanding how some features of QG are ontologically continuous to features of quantum mechanics. Second, it sets the stage for asking new interesting questions about QG, for instance concerning the status of the so-called Eigenstate-Eigenvalue link. Finally, it also indirectly shows how the debate on *ontological indeterminacy* may extend well beyond the non-relativistic case, contrary to what has been assumed so far.

## Data Availability

No datasets were generated or analysed during the current study.

## Footnotes

1 This is intended as an introductory statement, and we acknowledge that the status of spacetime in perturbative string theory is more nuanced than described here. A detailed discussion of the status of spacetime in perturbative string theory will be provided in Sect. 3.2. In particular, we will elaborate on how position and momentum are operators defined on the worldsheet but correspond to and are interpreted as the position and momentum of the string’s center of mass in the target space.

2 One might suggest that there is a third option here, namely spacetime being indeterminate and non-fundamental. This view, however, does not change our main point regarding the indeterminacy of spacetime, i.e. that the quantum properties of spacetime in QG can be recast in terms of indeterminacy rather than non-fundamentality. Hence, we will not discuss this option, as it would complicate our discussion unnecessarily by intertwining notions of quantumness, indeterminacy, and non-fundamentality that we prefer, for exposition purpose, to keep distinct via the two views contrasted in the main body of the paper.

3 Though note than some, most notably [15], disagree that EEL is part of the standard formalism. See [16] for a defence of EEL as part of standard QM.

4 See also: [1, 3, 5, 11, 13, 17–20]. For a critique, see [21], while, for an extensive review of the debate, see [10].

5 Most prominently, in the case of spontaneous collapse models, there have been several proposals on how to revise the EEL, all of which retain some indeterminacy. Examples include [25]’s *fuzzy link* [26]’s *vague link*, and [6]’s *degree link*.

6 As a matter of fact, some people have argued that the notion of *location* if correctly modified to account for quantum phenomena, may be useful as a replacement for the notion of indeterminacy. [REDACTED] This, we contend, can only be true if the *locations* themselves are not indeterminate.

7 If the reader does not like talk of measurements in this context, they can remove measurement from the discussion and think about there being a minimal length. The minimal length would be encoded in the spectrum of appropriate geometric operators, such as operators encoding lengths or areas, like the ones we discuss in what follows. The same disclaimer applies to all other occurrences of operational language in this article.

8 For discussion of this point, see [27, 30–34]. For philosophical discussion of QG theories with a minimal length and Lorentz invariance, see, e.g., [35].

9 Note that one might object here that there is no real contrast between Lorentz invariance and a minimal length, since the Planck length at least is defined in terms of Lorentz invariant quantities. While we agree with the spirit of this observation, we should clarify that our goal here is to show how indeterminacy arises in LQG via the tool of an apparent contrast between Lorentz invariance and minimal length. We do not wish to claim that such contrast is inevitable, but only that thinking about it leads naturally to recognize how indeterminacy can appear in LQG.

10 Indeed, boosts are Lorentz transformations not involving spatial rotations.

11 Note that, length contraction and time dilation are relative to a choice of clock synchrony convention. For a detailed discussion on this point, see [36].

12 We are bracketing potential issues regarding the practical feasibility of such measurements.

13 Indeed, this is the approach taken in [27] to analyse the apparent contradiction between Lorentz invariance and minimal length and show that it does not arise in Loop Quantum Gravity.

14 Not to be confused with the $\gamma$ in the Lorentz boosts.

15 We follow here usual practice in treating measurements as projections and bypass worries about the measurement problem, as they will not be relevant to our discussion here.

16 Which has the same spectrum of $\mathcal {A}$.

17 Modulo various technical subtleties which are not relevant for the argument in this section.

18 Let us briefly clarify what is meant by this point. Perturbative string theory differs from perturbative QFT in significant ways. While perturbative QFT deals with quantum fields spread over spacetime, perturbative string theory is more naturally analogous to the quantum mechanics of a worldline, generalized to a worldsheet. In QFT, the free theory is analytic and provides exact solutions without a perturbative expansion, while interactions are introduced perturbatively, often using Feynman diagrams. These expansions can be understood as conceptually equivalent to defining the theory on a worldline. In string theory, this framework extends to a worldsheet, creating a natural geometric interpretation. Extensions such as string field theory aim to align more closely with traditional field-theoretic approaches but have their own challenges. Additionally, frameworks like M-theory aim to provide a unified and non-perturbative description of interactions, which remains a central goal in the broader study of string theory.

19 For a book-length treatment, see [29], on which we draw for our discussion.

20 One needs to add fermions on the worldsheet by enforcing supersymmetry.

21 For a different approach, involving the non-perturbative sector of String Theory and with significant technical challenges, see [42]. These cases delve into non-commutative geometry and involve exact quantities that emerge in the non-perturbative description of string geometry, which are intrinsically non-perturbative in nature. While these topics raise fascinating questions about the role of indeterminacy in non-perturbative phenomena, we limit ourselves in this article to the perturbative sector for simplicity and clarity. We plan to explore these non-perturbative aspects in greater detail in future work.

22 The Polyakov action is chosen because it agrees with the Nambu-Goto action on-shell and has better behavior during quantization, making it a more convenient starting point for the quantum theory.

23 This is why we do not consider the worldsheet metric below.

24 Note that, we adopt the BRST approach to quantisation, which is a precise and systematic method for performing covariant quantization. This approach ensures consistency with the symmetries of the theory and is widely used in string theory. While other approaches, such as light-cone quantization, exist, they are non-covariant and often less systematic mathematically, making them harder to interpret conceptually. However, all valid quantisation schemes are expected to be equivalent to the covariant approach and, therefore, to the BRST formalism.

25 The conformal gauge is the gauge in which the worldsheet metric $h_{\alpha \beta }$ is flat, i.e. $h_{\alpha \beta }=\delta _{\alpha \beta }$. The following is also written in a new set of worldsheet coordinates: $\omega = t+i\sigma$ and $\bar{\omega }= t-i\sigma$ and $(\tau , \sigma ) \rightarrow (\omega ,\bar{\omega })$.

26 The momentum density *p* in analytic mechanics is defined as the functional derivative of the Lagrangian with respect to the temporal derivative of *q*, i.e. $p := \frac{\partial \mathcal L}{\partial \dot{q}}$, which generalises to our setup as $\mathcal P_\mu := \frac{\partial \mathcal L}{\partial \partial _t X^\mu }$.

27 The choice of the vacuum state $|0,P\rangle$ and the properties of this Hilbert space go beyond the scope of this paper.

28 A similar interpretation of the modes of the expansion also exists for open strings.

29 Note that by this we do not wish to commit ourselves to any specific ontology for quantum field theory. Our observation could easily be recast, from a field language, to the language of a particle interpretion.

30 As a reviewer pointed out, this could be explained by the fact that many people may agree with Wallace and simply reject the EEL. This is certainly a possibility, and we won’t enter into a detailed discussion of Wallace’s argument. We just notice that the EEL has been thoroughly discussed in the context of quantum mechanics (either to defend, or to reject it), while this hasn’t happened in QG. Either the status of the EEL changes in QG with respect to QM, in which case a discussion would be needed, or it doesn’t, in which case the overall assumption of our manuscript wouldn’t be totally justified. We thank the reviewer for inviting us to say more on this.

31 Once again, bracketing here concerns such as those of [15].

32 Wallace himself, who’s notoriously critical of the EEL, while discussing the *bare tails problem* claims the following: “One might be inclined to respond: so much the worse for the eigenvector-eigenvalue link, at least in the context of continuous observables. As we have seen [...] its motivation in modern QM is tenuous at best. But that simply transfers the problem: if the eigenvector-eigenvalue link is not to be the arbiter for which physical states count as localised, what is?” [43, p. 43]. This suggests that he himself agrees that something must be said, and right after this passage, in fact, he discusses Albert and Loewer *fuzzy link* as a way out. We think this shows, once again, that *something like the EEL* is needed to explain precisely how, from a delocalised wavefunction in space, we get to something close to an eigenstate of the position operator. We thank the reviewer for inviting us to add something here.

33 We want to thank an anonymous reviewer of this journal for inviting us to be more explicit.

34 For an example of an explicit construction of the algebra of operators for QG, see [44]. Lack of global symmetries has been shown explicitly, in the context of AdS/CFT, by [45]. The general case is a still-unproven but highly plausible constraint on any consistent theory of QG [46].

35 Here we assume the existence of an appropriate bridge principle between regions and spacetime which allows transferring indeterminacy from the former to the latter. While there is debate in the literature concerning the specific form of such a principle, with, for example, some arguing that *regions are nowhere because they are spacetime* [51] and others assuming that *regions are located in themselves* [52], we take it that any possible candidate will deliver the inference made here.

36 That regions must be determinate seems to be a standard, though maybe unsaid, assumption in the literature on theories of location. For an explicit argument, take for example the intuitive characterization of weak location of a given object *x* as a place *R* that is not completely free of *x*. This definition makes sense only if *R* is determinate, for otherwise there would be no fact of the matter as to whether certain objects, suitably located close to *R*’s boundary, would occupy *R*.

37 We believe it is quite natural to expect that an explanation of what an indeterminate spacetime region ultimately is, will depend on the details of the theory of ontological indeterminacy we assume in the background, and for instance whether we adopt a *meta-level* or an *object-level* approach.

38 For the rest of this section, we will broadly speak of spacetime, without distinguishing various ways and features of spacetime that might or might not be emergent, along the lines of the *spacetime quietism* advocated by [54]. However, some, most notably [55], have argued that such distinctions are crucial to properly analyze spacetime emergence. Given the broadly exploratory character of this section, we will continue, for ease of exposition, to simply talk of spacetime and eschew finer distinction.

39 Here, $\hbar = 0$ is used to conceptually indicate the classical limit, where quantum effects become negligible, rather than implying that the value of a fundamental constant can physically change. This phrasing identifies a regime in which quantum phenomena are suppressed, and for practical purposes, the system behaves classically. To formalize this, one could introduce a deformation parameter $\alpha$, such that $\alpha \hbar$ varies from 0 (classical limit) to $\hbar$ (fully quantum). This approach, which does not alter the value of $\hbar$, provides a more precise framework to describe the emergence of classical behavior.

40 For a review of decoherence, see [56]. For its use in the philosophical literature on quantum mechanics, in particular in the context of the quantum-classical transition, see [57, 58]; for applications of decoherence to the measurement problem, see [59, 60].

41 This observation is especially true once we consider the large literature developed on this topic in the context of QM, which could be then brought to bear directly on QG within the MI approach we are suggesting.

42 As shown, among others, by [52, 63, 64], the consequences of the emergent spacetime hypothesis may be potentially disruptive for many standard philosophical views in mereology, theories of causation, and formal views of location. These are, we believe, reasons enough to look for an alternative view such as the one we suggest in this paper.

43 Note that it is not clear that the two approaches, the *emergent* and the *indeterminate* spacetime, must be regarded as incompatible. For example, non-perturbative approaches to string theory like M-Theory, or AdS/CFT and holographic duality (note that we group AdS/CFT with non-perturbative approaches to string theory, since AdS/CFT is naturally understood as providing a non-perturbative completion for string theory in AdS via the dual CFT) might naturally incorporate both perspectives, given the more radical form of spacetime emergence involved. We leave these issues to future work.

## References

[1] Darby, G.: Quantum mechanics and metaphysical indeterminacy. Australas. J. Philos. **88**(2), 227–245 (2010)

[2] Skow, B.: Deep metaphysical indeterminacy. Philos. Q. **60**(241), 851–858 (2010)

[3] Bokulich, A.: Metaphysical indeterminacy, properties, and quantum theory. Res. Philos. **91**(3), 449–475 (2014)

[4] Calosi, C., Wilson, J.: Quantum metaphysical indeterminacy. Philos. Stud. **176**(10), 2599–2627 (2019)

[5] Schroeren, D.: Quantum metaphysical indeterminacy and the ontological foundations of orthodoxy. Stud. Hist. Philos. Sci. A **90**, 235–246 (2021) 10.1016/j.shpsa.2021.09.008 34740147

[6] Mariani, C.: Non-accessible mass and the ontology of grw. Stud. Hist. Philos. Sci. **91**, 270–279 (2022) 34999560 10.1016/j.shpsa.2021.11.015

[7] Calosi, C., Wilson, J.: Metaphysical indeterminacy in the multiuniverse. In: Allori, V. (ed.) Quantum Mechanics and Fundamentality. Springer, Cham (2021)

[8] Calosi, C., Mariani, C.: Quantum relational indeterminacy. Stud. Hist. Philos. Sci. B Stud. Hist. Philos. Mod. Phys. **71**, 158–169 (2020)

[9] Calosi, C.: Quantum modal indeterminacy. Stud. Hist. Philos. Sci. **95**, 177–184 (2022) 36084361 10.1016/j.shpsa.2022.08.012

[10] Calosi, C., Mariani, C.: Quantum indeterminacy. Philos. Compass **16**(4), e12731 (2021)

[11] Wolff, J.: Spin as a determinable. Topoi **34**(2), 379–386 (2015)

[12] Wüthrich, C., Le Bihan, B., Huggett, N.: Philosophy Beyond Spacetime: Implications from Quantum Gravity. Oxford University Press, Oxford (2021)

[13] Fletcher, S.C., Taylor, D.E.: Quantum indeterminacy and the eigenstate-eigenvalue link. Synthese **199**(3), 11181–11212 (2021)

[14] Huggett, N., Wüthrich, C.: Emergent spacetime and empirical (in) coherence. Stud. Hist. Philos. Sci. B Stud. Hist. Philos. Mod. Phys. **44**(3), 276–285 (2013)

[15] Wallace, D.: What is orthodox quantum mechanics? In: Philosophers Look at Quantum Mechanics, pp. 285–312. Springer, Cham (2019)

[16] Gilton, M.J.: Whence the eigenstate-eigenvalue link? Stud. Hist. Philos. Sci. B Stud. Hist. Philos. Mod. Phys. **55**, 92–100 (2016)

[17] Darby, G.: Vague objects in quantum mechanics? In: Vague Objects and Vague Identity, pp. 69–108. Springer, Cham (2014)

[18] Torza, A.: Quantum metaphysical indeterminacy and worldly incompleteness. Synthese **197**(10), 4251–4264 (2020)

[19] Corti, A.: Yet again, quantum indeterminacy is not worldly indecision. Synthese **199**(3), 5623–5643 (2021)

[20] Mariani, C., Michels, R., Torrengo, G.: Plural metaphysical supervaluationism. Inquiry **67**(6), 2005–2042 (2024)

[21] Glick, D.: Against quantum indeterminacy. Thought A J. Philos. **6**(3), 204–213 (2017)

[22] Wilson, J.: Are there indeterminate states of affairs? yes. In: Current Controversies in Metaphysics, pp. 105–119. Routledge, Milton Park (2016)

[23] Wilson, J.M.: A determinable-based account of metaphysical indeterminacy. Inquiry **56**(4), 359–385 (2013)

[24] Calosi, C.: Gappy, glutty, glappy. Synthese **199**(3), 11305–11321 (2021)

[25] Albert, D.Z., Loewer, B.: Tails of Schrödinger’s cat. In: Perspectives on Quantum Reality, pp. 81–92. Springer, Cham (1996)

[26] Lewis, P.J.: Quantum Ontology: A Guide to the Metaphysics of Quantum Mechanics. Oxford University Press, Oxford (2016)

[27] Rovelli, C., Speziale, S.: Reconcile planck-scale discreteness and the lorentz-fitzgerald contraction. Phys. Rev. D **67**(6), 064019 (2003)

[28] Livine, E.R., Oriti, D.: About Lorentz invariance in a discrete quantum setting. J. High Energy Phys. **2004**(06), 050 (2004)

[29] Green, M.B., Schwarz, J.H., Witten, E.: Superstring Theory. Cambridge University Press, Cambridge (1987)

[30] Amelino-Camelia, G.: Testable scenario for relativity with minimum length. Phys. Lett. B **510**(1–4), 255–263 (2001)

[31] Amelino-Camelia, G.: Relativity in spacetimes with short-distance structure governed by an observer-independent (planckian) length scale. Int. J. Mod. Phys. D **11**(01), 35–59 (2002)

[32] Magueijo, J., Smolin, L.: Lorentz invariance with an invariant energy scale. Phys. Rev. Lett. **88**(19), 190403 (2002) 12005620 10.1103/PhysRevLett.88.190403

[33] Amelino-Camelia, G.: The three perspectives on the quantum-gravity problem and their implications for the fate of lorentz symmetry. In: The Tenth Marcel Grossmann Meeting: On Recent Developments in Theoretical and Experimental General Relativity, Gravitation and Relativistic Field Theories (In 3 Volumes), pp. 2214–2216. World Scientific (2005)

[34] Smolin, L.: Falsifiable predictions from semiclassical quantum gravity. Nucl. Phys. B **742**(1–3), 142–157 (2006)

[35] Hagar, A.: Minimal length in quantum gravity and the fate of lorentz invariance. Stud. Hist. Philos. Sci. B Stud. Hist. Philos. Mod. Phys. **40**(3), 259–267 (2009)

[36] Brown, H.: Physical Relativity: Space-Time Structure from a Dynamical Perspective. Oxford University Press, Oxford (2005)

[37] Rovelli, C.: Covariant Loop Quantum Gravity: An Elementary Introduction to Quantum Gravity and Spinfoam Theory. Cambridge University Press, Cambridge (2004)

[38] Rovelli, C., Vidotto, F.: Covariant Loop Quantum Gravity: An Elementary Introduction to Quantum Gravity and Spinfoam Theory. Cambridge University Press, Cambridge (2014)

[39] Maldacena, J.: The large-N limit of superconformal field theories and supergravity. Int. J. Theor. Phys. **38**(4), 1113–1133 (1999)

[40] Witten, E.: String theory dynamics in various dimensions. Nucl. Phys. B **443**(1–2), 85–126 (1995)

[41] Vafa, C.: Evidence for F-theory. Nucl. Phys. B **469**(3), 403–415 (1996)

[42] Seiberg, N., Witten, E.: String theory and noncommutative geometry. J. High Energy Phys. **1999**(09), 032 (1999)

[43] Wallace, D.: The quantum measurement problem: state of play. In: Rickles, D. (ed.) The Ashgate Companion to Contemporary Philosophy of Physics. Cornell University, Ithaca (2008)

[44] Harlow, D., Wu, J.-Q.: Algebra of diffeomorphism-invariant observables in Jackiw-Teitelboim gravity. J. High Energy Phys. **2022**(5), 1–91 (2022) 10.1007/JHEP05(2022)097 PMC9113916 35602930

[45] Harlow, D., Ooguri, H.: Symmetries in quantum field theory and quantum gravity. Commun. Math. Phys. **383**(3), 1669–1804 (2021)

[46] Vafa, C., Brennan, T.D., Carta, F.: The string landscape, the swampland, and the missing corner. PoS TASI **2017**, 015 (2018)

[47] Andringa, R., Bergshoeff, E., Panda, S., De Roo, M.: Newtonian gravity and the Bargmann algebra. Class. Quantum Gravity **28**(10), 105011 (2011)

[48] Lam, V., Letertre, L., Mariani, C.: Quantum metaphysics and the foundations of spacetime. In: Vassallo, A. (ed.) The Foundations of Spacetime Physics: Philosophical Perspectives, pp. 371–393. Routledge, Milton Park (2022)

[49] Calosi, C.: Determinables, location, and indeterminacy. Synthese **198**(5), 4191–4204 (2021)

[50] Calosi, C., Wilson, J.: Quantum indeterminacy and the double-slit experiment. Philos. Stud. **178**(10), 3291–3317 (2021)

[51] Casati, R., Varzi, A.C., et al.: Parts and Places: The Structures of Spatial Representation. MIT Press, Cambridge (1999)

[52] Baron, S.: The curious case of spacetime emergence. Philos. Stud. **177**(8), 2207–2226 (2020)

[53] Parsons, J.: Theories of location. Oxford studies in metaphysics **3**, 201–232 (2007)

[54] Baron, S., Le Bihan, B.: Spacetime quietism in quantum gravity. In: The Foundations of Spacetime Physics, pp. 155–175. Routledge, Milton Park (2022)

[55] Jaksland, R., Salimkhani, K.: The many problems of spacetime emergence in quantum gravity. Br. J. Philos. Sci. (2023). 10.1086/727052

[56] Schlosshauer, M.: Quantum decoherence. Phys. Rep. **831**, 1–57 (2019)

[57] Rosaler, J.: Formal versus empirical approaches to quantum-classical reduction. Topoi **34**(2), 325–338 (2015)

[58] Rosaler, J.: Interpretation neutrality in the classical domain of quantum theory. Stud. Hist. Philos. Sci. B: Stud. Hist. Philos. Mod. Phys. **53**, 54–72 (2016)

[59] Wallace, D.: Decoherence and its role in the modern measurement problem. Philos. Trans. Royal Soc. A Math. Phys. Eng. Sci. **370**(1975), 4576–4593 (2012) 10.1098/rsta.2011.0490 22908343

[60] Bacciagaluppi, G.: The Role of Decoherence in Quantum Mechanics. In: Zalta, E.N. (ed.) The Stanford Encyclopedia of Philosophy. Metaphysics Research Lab, Stanford University, Stanford (2020)

[61] Le Bihan, B.: Spacetime emergence in quantum gravity: functionalism and the hard problem. Synthese **199**(Suppl 2), 371–393 (2021)

[62] Huggett, N., Wüthrich, C.: Out of Nowhere: The Emergence of Spacetime in Quantum Theories of Gravity. Oxford University Press, Oxford (2024)

[63] Baron, S., Miller, K., Tallant, J.: Out of Time. Oxford University Press, Oxford (2022)

[64] Baron, S., Le Bihan, B.: Quantum gravity and mereology: not so simple. Philos. Q. **72**(1), 19–40 (2022)

